# Unhealthy life habits associated with self-induced vomiting and laxative misuse in Brazilian adolescents

**DOI:** 10.1038/s41598-021-81942-w

**Published:** 2021-01-28

**Authors:** Ana Luiza Gomes de Souza, Alexandre Aparecido de Almeida, Priscilla Rayanne E. Silva Noll, Matias Noll

**Affiliations:** 1 Instituto Federal de Educação, Ciência e Tecnologia Goiano, Campus Ceres, Ceres, GO Brazil; 2 Instituto Federal de Educação, Ciência e Tecnologia do Tocantins, Campus Araguatins, Araguatins, TO Brazil; 3 Department of Obstetrics and Gynecology, Faculdade de Medicina FMUSP, Universidade de São Paulo, São Paulo, SP Brazil

**Keywords:** Nutrition disorders, Risk factors

## Abstract

Adolescence is a stage in life characterized by important social, cognitive, and physical changes. Adolescents are vulnerable to various psychosocial disorders, including eating disorders. We aimed to investigate the association between unhealthy habits, sociodemographic characteristics, and the practice of self-induced vomiting or laxative misuse in a representative sample of Brazilian adolescent girls and boys. Data from 102,072 students who participated in the National Adolescent School-based Health Survey were analyzed using the dependent variable: presence or absence of self-induced vomiting and/or laxative misuse; independent variables: consumption of unhealthy and high-calorie food items, age during first sexual intercourse, and the use of tobacco, alcohol, and/or illicit drugs. Associations between exposure and outcome were estimated using Poisson’s regression models stratified by sex, and including region, school, age group, and mother's educational history as adjustment variables. Eating ultra-processed foods and age during first sexual intercourse were associated with self-induced vomiting and laxative misuse only for girls; all other variables (consuming unhealthy foods and using legal or illicit substances) were associated with these behaviors for both sexes after applying adjustment variables. Early interventions focusing on changing unhealthy behaviors may prevent development of eating disorders in adolescents*.* Our findings demonstrate a strong association of many unhealthy habits with laxative misuse and self-induced vomiting practices in Brazilian adolescents.

## Introduction

Adolescence, commonly considered to occur from ten to nineteen years old^1^, is a period in life characterized by important social, cognitive, and physical changes for young people^2^. In particular, adolescents undergo considerable corporal changes such as increasing body mass and stature, body hair growth, voice thickening, and the emerging of secondary sexual characteristics^2,3^. In addition to these physical changes, they are also developing psychologically, making them vulnerable to various psychosocial disorders, including eating disorders (EDs).

EDs can be considered psychiatric conditions with serious medical and psychological consequences^4^. In general, they are characterized by gross disturbances in eating behavior as well as extreme and distorted concerns about body shape and/or weight^5^. EDs are disorders involving disordered eating, such as anorexia nervosa, bulimia nervosa, binge eating, orthorexia, and avoidant restrictive food intake disorder (ARFID)^4,6^. Recent studies^7^ have shown that factors contributing to the development of EDs are diverse and complex with varying symptoms. The most detrimental symptoms of EDs could vary across individuals (as fear of weight gain and feelings of fatness) and within individuals (as excessive exercise, fear of weight gain, and feelings of fatness). Studies also reveal several significant factors often associated with EDs, such as: body dissatisfaction^8,9^, thin-ideal internalization^8,9^, dieting^8^, overeating^8^, negative feelings (sadness, guilt, and fear/anxiety)^8,10^, functional impairment^8^, mental health^8^, neurocognitive functioning^11^, early traumatic and stressful events, sexual abuse^12^, parental and peer influences^13^, and level of physical activity^12^. Moreover, body changes during the transition from childhood to adolescence^3,14^ may be related to the increasing rates of overweight in adolescents^15,16^. Physical appearance standards promoted in the media potentially influence developing EDs, when associated with feelings of obesity and fear of weight gain^17–19^, which are primary symptoms of EDs^7^.

From a health viewpoint, previous studies in adults have demonstrated that both anorexia nervosa and bulimia nervosa are associated with inappropriate compensatory behaviors such as self-induced vomiting and laxative misuse^20^. North American adolescents report high prevalence of body shape dissatisfaction; it is postulated that about 58.8% of girls and 37.4% of boys make use of unhealthy methods to control weight, including fasting, diet pills, self-induced vomiting, dietary restraint, food substitutes (special drinks or powder), skipping meals, smoking more cigarettes, and other purging practices such as laxatives and diuretics^21^. For instance, in a non-eating-disordered sample of female high school students from the United States, Phelps and colleagues (1993)^22^ found a prevalence of 3.2–5.5% for laxative misuse and 13.5–6.1% for self-induced vomit practices^22^. Furthermore, Westenhoefer (2001)^23^ verified there was a prevalence ranging between 0.7–4.7% for diuretic/laxative misuse and 0.5–1.6% for vomiting in a German sample of an adult population. This prevalence was much higher in adolescent and adult patients being clinically treated for a diagnosed ED, for whom the prevalence ranged from 4–35% and 11%–63% in anorexia nervosa and bulimia nervosa, respectively^24–27^. Hence, disordered weight-control behaviors in youth are a growing concern and they often begin prior to the onset of EDs^28,29^. As these methods have important negative implications for health^30,31^, identifying factors that lead individuals to use them and providing conditions for people to avoid these practices are important aspects of preventive medicine strategies.

Unhealthy habits such as consuming alcohol appear to co-exist with inappropriate compensatory behaviors. For instance, college students report the practice of self-induced vomiting, excessive exercising, and the misuse of diuretics, laxatives, and diet pills to counteract the effects of alcohol consumption on weight gain^32,33^. Additionally, a considerable number of college-aged youth report restricting their caloric intake before drinking to prevent weight gain^32–36^. Thus, alcohol misuse may be a risk factor for inappropriate compensatory behavior and posterior eating disorder development. As unhealthy life habits evolving from the promotion and sustained abuse of inappropriate compensatory methods for weight loss in adolescents are not completely understood, the present study aimed to investigate the association between sociodemographic characteristics and unhealthy life habits with the practices of self-induced vomiting or laxative misuse in a representative sample of Brazilian adolescents.

Our study evaluated several unhealthy life habits, such as smoking; alcohol use or alcohol binging; illicit drugs use; overconsumption of ultra-processed salty foods, sweets, and soft drinks; and age during first sexual intercourse. Although a systematic review showed that EDs are a common mental disorder in Latin America (Argentina, Brazil, Chile, Colombia, Mexico, and Venezuela), there is a lack of evidence indicating the factors associated with EDs in representative samples of adolescents^37^. Epidemiological research investigating the factors associated with EDs^38^ using population-based studies is scarce in Brazil^39^. The literature highlights that the prevalence of EDs in specific Brazilian cities^38–40^ varies between 8.3–17.3%. Pivetta and Gonçalves-Silva^39^ demonstrate high prevalence of binge eating episodes associated with female gender, age, alcohol consumption, and weight oscillation in adolescents attending high school in Brazil. Therefore, our study focused on filling gaps in the previous literature pertaining to the lack of representation in certain areas of Latin America; thus, presenting results from a representative sample of Brazilian adolescents.

## Methods

### Data sources and study population

This study used data from the 2015 PeNSE^41^ survey, which assessed students aged 11–19 classified as 9th graders from both public and private schools throughout Brazil. This umbrella study was conducted by a partnership between the Ministry of Health and the Brazilian Institute of Geography and Statistics (Instituto Brasileiro de Geografia e Estatística; IBGE)^42^.

The sample size calculations were based on parameters for all 26 states and the Federal District, including within each state, the capital, and other municipalities with smaller populations. Brazil is conventionally divided into five regions: South, North, Northeast, Southeast, and Midwest^42^. Samples across capitals and municipalities were random and equiprobabilistic. The following parameters were used for deriving the sample: maximum error of 3%, confidence level of 95%, and prevalence of 0.5. All students in the sample classes were invited to respond to the survey questionnaire. Further information on sampling procedures may be found in Oliveira et al.^42^.

The PeNSE acquired consent from the heads of the schools and all students, who voluntarily agreed to participate by providing written informed consent. Students could leave the study at any time and could refuse to participate during the assessment. Anonymity and privacy were fully guaranteed to the participants. This study was conducted in accordance with the Declaration of Helsinki and was approved by the National Commission on Ethics in Research of the National Health Council, which regulates and approves health research involving human participants (no. 1,006,467)^41,42^.

A total of 120,122 students, attending 4159 classes across 3040 schools, were included in the sample in 2015. From all students enrolled, 15% of regular students did not attend the class, did not agree to participate, or did not fully complete the questionnaire. A total of 102,072 students completed the survey questionnaire on the sampling day.

### Data collection and data analysis

PeNSE data collection assesses various health outcomes and risk behaviors and several studies have been dedicated to assessing these aspects^43–49^. Data collection was performed using smartphones distributed to students who were in class on the day of the interview by an IBGE technician. The technician explained how to use the device and was available to help the students when required^42^.

The outcome was laxative misuse and/or self-induced vomiting practice, which was assessed by the following question: Have you vomited or taken laxatives to lose weight or avoid gaining weight? Answer options were yes and no.

The evaluated health-risk behavior explanatory variables were: tobacco use (smoked at least one prior time; frequency of smoking over the past 30 days); alcohol use (experienced drinking at least once; frequency of drinking over the past 30 days; experienced getting drunk at least once); illegal drug use (marijuana, cocaine, crack [used at least one prior time; frequency of use over the past 30 days]); sexual behavior (age during first sexual intercourse); and regular consumption of high-calorie ultra-processed food (ultra-processed salty food, sweets, and soft drinks).

Food consumption was categorized as regularly consumed [at least 5 days in the previous 7 days] or not regularly consumed [less than 5 days in the previous 7 days] for data analysis^50,51^.

The adjustment variables included sociodemographic characteristics*:* region (South, North, Northeast, Southeast, and Midwest), school (public or private), sex, age group, and mother's educational history (none, elementary school, high school, graduate or higher).

### Statistical analysis

The data for self-induced vomiting and/or laxative misuse (dependent variables) were analyzed through descriptive statistics (absolute and percentage) and through the Wald chi-squared test (bivariate analysis). The following factors were considered independent variables: tobacco use, alcohol use, illegal drug use, sexual behavior, and processed high-calorie food items. The independent variables were analyzed using the Poisson regression model with robust variance (crude analysis). The effect measure was the prevalence ratio (PR) with its respective 95% confidence intervals (α = 0.05)^52^. Crude PR was obtained by unadjusted Poisson regression.

We also performed adjusted regression analyses by the Poisson model, being the results adjusted by sociodemographic variables (region, school, age group, and mother's schooling). These adjustment variables were selected based on potential confounding factors supported by methodological and statistical studies^52–54^. We performed the analysis separately for each sex because adjusting the model by sex might not have provided reliable findings. The effect measure was the adjusted PR (Adj PR) with its respective 95% confidence intervals (α = 0.05)^52^. All analyses were conducted using the Statistical Package for the Social Sciences (SPSS 26.0, IBM, Armonk, NY, USA).

## Results

The total sample included 102,072 students. The majority of the subjects were female (n = 52,782; 51.7%), subjects’ average age was 14 years (SD = 1.06; range = 11–19) and most were enrolled in public schools (n = 81,154; 79.5%). The Northeast region of Brazil provided the largest proportion of students in the sample (n = 36,334; 35.6%), followed by the North with 23.5% (n = 23,937). Of all volunteers, 7041 (6.9%) reported having used any compensatory behavior to lose weight (6.4% for boys and 7.5% for girls) (Table 1).

**Table 1:** Sociodemographic characteristics of participants as related to laxative misuse and/or self-induced vomiting practice.

Variables	Total		Laxative misuse and/or self–induced vomiting practice		
	N	%	N	%	*p*
**Sex**					
Male	49,290	48.3	3097	6.4	< 0.001
Female	52,782	51.7	3944	7.5	
**Region**					
North	23,937	23.5	1965	8.3	< 0.001
Northeast	36,334	35.6	2292	6.4	
Southeast	17,772	17.4	1109	6.3	
South	9850	9.7	620	6.4	
Midwest	14,179	13.9	1055	7.5	
**School**					
Public	81,154	79.5	5827	7.3	< 0.001
Private	20,918	20.5	1214	5.8	
**Age group**					
≤ 13 years	17,260	16.9	911	5.3	< 0.001
14 years	51,611	50.6	3212	6.3	
15 years	20,864	20.4	1662	8.1	
> 16 years	12,337	12.1	1256	10.4	
**Mother's education**					
None	5531	7.2	596	11.0	< 0.001
Elementary School^a^	24,241	31.6	1757	7.3	
High School^a^	24,178	31.5	1557	6.5	
Graduate^a^	22,688	29.6	1537	6.8	

^a^Complete and incomplete educational level.

For both sexes, we observed frequencies of smoking (number of days having smoked in the last 30 days), drinking (number of days drinking and number of days drinking until drunk), illicit drug use (number of days illicit drugs were used in the last 30 days), consumption of soft drinks in the last seven days, and consumption of sweets in the last seven days were associated to self-induced vomiting and laxative misuse. Consumption of ultra-processed salty food in the last seven days and age during first sexual intercourse were associated with self-induced vomiting and laxative misuse only for girls (Table 2). After adjusted analysis, the associations regarding the variables remained the same (Table 3).

**Table 2:** Association between unhealthy life habits and laxative misuse and/or self-induced vomiting in Brazilian adolescent boys and girls (N = 102,072).

Variables	Male				Female			
	Total (%)	Laxative misuse and/or self-induced vomiting			Total (%)	Laxative misuse and/or self-induced vomiting		
		%	Crude PR (95% CI)	*p*		%	Crude PR (95% CI)	*p*
**Experience smoking**								
No	80.3	5.3	1	< 0.001	82.9	6.1	1	< 0.001
Yes	19.7	10.8	2.04 (1.9–2.19)		17.1	14.5	2.37 (2.23–2.52)	
Smoked during the last 30 days*								
None	70.7	8.3	1	< 0.001	71.3	12.3	1	< 0.001
1–2 days	14.3	14.9	1.8 (1.54–2.09)		16.7	16.9	1.38 (1.21–1.57)	
3–9 days	7.3	14.8	1.79 (1.47–2.17)		6.8	21.3	1.73 (1.47–2.05)	
10 or more	7.7	22.5	2.72 (2.32 – 3.18)		5.2	27.5	2.24 (1.91–2.63)	
**Experience drinking**								
No	49.2	4.8	1	< 0.001	47.4	4.4	1	< 0.001
Yes	50.8	8.0	1.68 (1.56–1.8)		52.6	10.3	2.33 (2.18–2.49)	
Drinking during the last 30 days*								
None	60.1	6.0	1	< 0.001	57.7	8.2	1	< 0.001
1–2 days	23.2	9.6	1.59 (1.44–1.75)		26.8	10.7	1.31 (1.2–1.42)	
3–9 days	11.3	11.5	1.91 (1.7–2.15)		10.9	15.8	1.93 (1.75–2.12)	
10 or more	5.4	18.5	3.07 (2.7–3.48)		4.6	20.5	2.5 (2.2–2.82)	
Experience getting drunk*								
None	61.2	6.2	1	< 0.001	63.7	8.0	1	< 0.001
1–2 days	24.1	9.3	1.49 (1.35–1.64)		25.3	12.4	1.55 (1.43–1.68)	
3–9 days	9.5	13.1	2.11 (1.87–2.37)		8.0	16.4	2.05 (1.84–2.28)	
10 or more	5.2	16.4	2.64 (2.3–3.02)		3.1	23.4	2.93 (2.57–3.33)	
**Previous illicit drug use (marijuana, cocaine, crack)**								
No	90.7	5.7	1	< 0.001	92.3	6.7	1	< 0.001
Yes	9.3	13.3	2.34 (2.15–2.55)		7.7	17.2	2.57 (2.38–2.77)	
Used drugs in the last 30 days*								
None	52.7	10.9	1	< 0.001	56.6	15.0	1	< 0.001
1–2 days	22.3	14.8	1.36 (1.13–1.64)		24.0	19.1	1.28 (1.09–1.51)	
3–9 days	13.1	15.5	1.42 (1.14–1.77)		12.2	20.6	1.38 (1.13–1.68)	
10 or more	11.8	18.3	1.68 (1.35–2.08)		7.2	23.0	1.54 (1.22–1.94)	
**Consumption of ultra-processed salt food in the last 7 days**								
0–4 days	70.3	6.4	1	0.820	66.6	7.0	1	< 0.001
5–7 days	29.7	6.3	0.99 (0.92–1.07)		33.4	8.5	1.22 (1.15–1.3)	
**Consumption of soft drinks in the last 7 days**								
0–4 days	72.6	5.7	1	< 0.001	75.5	6.9	1	< 0.001
5–7 days	27.4	8.3	1.46 (1.36–1.57)		24.3	9.6	1.39 (1.31–1.49)	
**Consumption of sweets in the last 7 days**								
0–4 days	66.5	6.1	1	< 0.001	54.5	7.0	1	< 0.001
5–7 days	33.5	7.0	1.14 (1.06–1.22)		45.5	8.2	1.17 (1.1–1.24)	
Age of first sexual intercourse*								
≥ 15 years	15.9	10.5	1	0.089	25.3	11.5	1	0.001
13 and 14 years	50.3	9.9	0.94 (0.83–1.07)		62.1	11.8	1.03 (0.91–1.17)	
≤ 12 years	33.8	11.0	1.05 (0.92–1.19)		12.6	15.5	1.35 (1.15–1.6)	

The analysis was conducted using the Poisson regression model with robust variance. The effect measure is the PR with its respective 95% CI.

*Crude PR* unadjusted prevalence ratio, *95% CI* 95% confidence interval. * Only where applicable.

**Table 3:** Adjusted analysis between unhealthy life habits and laxative misuse and/or self-induced vomiting in Brazilian adolescent boys and girls (N = 102,072).

Variables	Male		Female	
	Adj PR (95% CI)	*p*	Adj PR (95% CI)	*p*
**Experience smoking**				
No	1	< 0.001	1	< 0.001
Yes	1.79 (1.65–1.96)		2.27 (2.1–2.44)	
Smoked during the last 30 days*				
None	1	< 0.001	1	< 0.001
1–2 days	1.72 (1.45–2.05)		1.39 (1.2–1.6)	
3–9 days	1.61 (1.28–2.03)		1.67 (1.38–2.01)	
10 or more	2.6 (2.16–3.11)		2.21 (1.84–2.66)	
**Experience drinking**				
No	1	< 0.001	1	< 0.001
Yes	1.59 (1.46–1.73)		2.27 (2.1–2.46)	
Drinking during the last 30 days*				
None	1	< 0.001	1	< 0.001
1–2 days	1.59 (1.42–1.79)		1.31 (1.2–1.44)	
3–9 days	1.95 (1.7–2.23)		1.8 (1.61–2)	
10 or more	2.93 (2.53–3.41)		2.41 (2.11–2.76)	
Experience getting drunk*				
None	1	< 0.001	1	< 0.001
1–2 days	1.46 (1.3–1.64)		1.53 (1.4–1.67)	
3–9 days	2.11 (1.84–2.42)		1.94 (1.72–2.19)	
10 or more	2.56 (2.18–3)		2.72 (2.34–3.17)	
**Previous illicit drug use (marijuana, cocaine, crack)**				
No	1	< 0.001	1	< 0.001
Yes	2.1 (1.9–2.32)		2.36 (2.16–2.58)	
Used drugs in the last 30 days*				
None	1	< 0.001	1	0.004
1–2 days	1.29 (1.04–1.6)		1.23 (1.03–1.48)	
3–9 days	1.48 (1.15–1.89)		1.35 (1.08–1.68)	
10 or more	1.64 (1.27–2.11)		1.44 (1.1–1.9)	
**Consumption of ultra-processed salt food in the last 7 days**				
0–4 days	1	0.051	1	< 0.001
5–7 days	1.09 (1–1.19)		1.19 (1.1–1.28)	
**Consumption of soft drinks in the last 7 days**				
0–4 days	1	< 0.001	1	< 0.001
5–7 days	1.47 (1.35–1.6)		1.31 (1.22–1.41)	
**Consumption of sweets in the last 7 days**				
0–4 days	1	0.001	1	< 0.001
5–7 days	1.15 (1.06–1.25)		1.15 (1.08–1.23)	
**Age of first sexual intercourse**				
≥ 15 years	1	0.183	1	0.011
13 and 14 years	1.01 (0.87–1.18)		0.93 (0.78–1.1)	
≤ 12 years	1.11 (0.95–1.31)		1.19 (1.02–1.36)	

The analysis was conducted using the Poisson regression model with robust variance. The effect measure is the PR with its respective 95% CI. The model was adjusted for confounding variables: region, school, age group, and mother's schooling.

*Adj PR* adjusted prevalence ratio, *CI* confidence interval. * Only where applicable.

## Discussion

The present study aimed to investigate the association between unhealthy life habits and the practice of self-induced vomiting or laxative misuse among Brazilian adolescents. Our main results showed several unhealthy life habits associated with the practice of self-induced vomiting or laxative misuse among Brazilian adolescents, with similar findings for both sexes. An exception is observed regarding early sexual intercourse and the number of days of consuming ultra-processed foods in boys. In these cases, only girls who had their first sexual relations before the age of 12 years and girls who ate ultra-processed foods between 5 and 7 days per week showed a higher prevalence of laxative misuse or self-induced vomiting practice.

In the present study, we observed a considerable prevalence of alcohol misuse in adolescents of both sexes. When alcohol misuse is present, laxative misuse and/or self-induced vomiting is 1.68 times higher for boys and 2.33 times higher for girls. Alcohol abuse in adolescence has serious negative personal consequences, such as poorer academic achievement and poor health outcomes^55^, and potentially leads to damaging the structure and functioning of the developing brain^56,57^, resulting in health-risk behaviors^58–60^ and associated extensive societal costs. Moreover, studies have pointed out that early-life psychoactive substance use, including alcohol consumption, correlates to higher levels of use and abuse of drugs in adulthood^61^. College students engaged in inappropriate compensatory behaviors before, during, and after drinking alcohol as a way to counteract the effects of alcohol consumption on weight gain^32,33,62^. Our results indicated that the chance of laxative misuse and/or self-induced vomiting is related to higher frequency of adolescent drinking per week as well as higher frequency of getting drunk, for both sexes.

Some studies pointed out a co-occurrence between binge drinking and problematic eating behavior, especially in relation to binge EDs in different populations^63–66^. Considering the previously-cited significant negative consequences directly related to adolescent binge eating and drinking behavior, both must be treated in the early stages of their development to provide an improvement in the quality of life and to reduce the chances of young people experiencing the deleterious effects of both.

More frequent smoking and using illicit drugs are related to a higher chance of laxative misuse and self-induce vomiting. Our results aligned with an observed tendency toward higher tobacco use in girls in the last decades^67^, and it is an unhealthy behavior used to avoid weight gain^68,69^, also supported by our data. In addition to the greater body dissatisfaction pointed out by girls, it is a common belief that smoking cigarettes has effects on weight control^69,70^. Furthermore, as substance use can exacerbate impulsivity, emotional instability, and other psychiatric symptoms, it may be especially dangerous for adolescents with EDs^71^. Finally, adding to the complexity of the relationships between smoking, illicit substance use, and EDs^72–75^, there appears to be a longstanding belief that nicotine and drug components suppress appetite, and smoking has been shown to increase resting metabolic rate^76^. This belief provokes some women affected by EDs to use smoking as a weight-control strategy^77^. This belief also contributes to individuals with ED’s perception that the benefits of nicotine for weight control and temporary stress reduction may outweigh any concerns about the long-term prejudicial effects of smoking^72,78^. Thus, identifying unhealthy behaviors before EDs are established appears to be the best strategy for helping young people avoid ED development and the associated negative consequences on their health.

Adolescents consume a large quantity of ultra-processed foods, such as soft drinks, sweets, and treats. The consumption of these food groups increased in adolescents with restrictive food intake disorder^79^. Additionally, eating ultra-processed foods is related to negative emotions, stress, and anxiety-induced sleep disturbance^80–83^. In this regard, our results show that the regular consumption of soft drinks and ingestion of sweets are significantly associated with laxative misuse and self-induced vomiting for both sexes, and ultra-processed foods consumption is associated with these behaviors in females. In a population of Brazilian university students, women dissatisfied with their body image, present in EDs, reported higher consumption of ultra-processed food than women satisfied with their body image (27.8% and 23.6%, respectively)^84^. Ultra-processed foods may facilitate overeating because they are typically high in calories, fat, sugar, and salt. Furthermore, it has been suggested that ultra-processed foods are engineered to have higher appetitive properties and may lead to compulsive food consumption^85–88^.

Our data demonstrate that having their first sexual intercourse before the age of 12 was associated with self-induced vomiting and laxative misuse in girls. The percentage of girls that had their first sexual intercourse was similar to that found in a study performed by Malta et al.^89^. Regardless of these results, early sexual intercourse is considered a general risky behavior related to various health and socioemotional problems. For instance, studies have demonstrated that men and women with early first sexual intercourse (< 16 years) were more likely to report risky sexual behavior without using a condom, more sex under the influence of drugs, and higher occurrences of sexually transmitted infections and teen pregnancy^90–93^. Studies indicate that age at first sexual intercourse is associated with tobacco and alcohol use, binge drinking, and illicit drug use^94–96^, but is not linked to the occurrence of EDs since the age of first sexual intercourse does not differ between women with or without EDs^94^. It is not yet possible to say with certainty which problem arises first in the lives of adolescents, and no causal relation can be stated due to the cross-sectional design of our study. Considering the serious negative impacts that drug use and risky sexual behavior have on adolescents' lives, it is critical for educational campaigns and public health policies directed at adolescents to focus on these issues.

Some limitations of this study must be pointed out. First, the PeNSE used surveys to record the data, and the questionnaire was developed specifically for the Brazilian National Survey. Surveys can introduce bias because not everybody is honest in answering them^97^. In this case, interview studies may be more adequate. In contrast, data collection by interview is more expensive and takes longer to get data from a single individual, making it unreasonable for populational studies. Second, we do not have separate analyses for laxative and diuretic misuse practices. As the two behaviors are different in concepts and effects on the body, conjunction analysis of two may have omitted important associations. Third, the use of cross-sectional data limits the ability to draw causal or developmental conclusions. Fourth, the fact for the Brazilian educational system is that 11.8% of youth between 15 and 17 years were not enrolled in any school in 2018 (more recent survey data); thus, our data are not representative of all persons in this age group, applying only to youths who attend school. Fifth, behaviors were mostly measured by a single question and data were self-reported, which may have been influenced by level of interpretation and cognition, and by social desirability biases. Sixth, we have not evaluated all the important weight-control variables related to ED, such as eating restriction, compulsive exercise, or fasting; each of these factors should be addressed in future populational studies. Seventh, the data does not include body mass index and ED diagnoses, because it is a self-administered questionnaire by students. Moreover, we suggest that future studies evaluate an important outcome, the ARFID^98^.

Our results bring new insights into the association of unhealthy behaviors and the use of laxatives and self-induced vomiting in adolescents. However, questions remain to be answered related to which changes arise first, the use of psychoactive substances or compensatory behaviors related to body-weight control. Furthermore, it is not providential that young people who use unhealthy methods to control weight will develop more serious EDs in the future and have higher risks of developing EDs. Lastly, a study using neuroimaging in a non-clinical population of adolescents could provide insights about minor brain alterations related to the development of EDs.

## Conclusion

Our findings demonstrate a strong association between many unhealthy habits and laxative misuse and self-induced vomiting practices in Brazilian adolescents of both sexes. Demonstration of concurrent relationships between unhealthy behavior and laxative misuse and self-induced vomiting practice among adolescents may provide insight into emphasis points for anticipatory guidance around risk and coping behaviors for youth, helping health professionals expand public politics to prevent the development of EDs. Considering that brain mechanisms involved in the use of alcohol and cigarettes and EDs are similar, mandatory educational interventions that seek to reduce both may indirectly contribute to avoiding more serious unhealthy attitudes. Providing understanding to teachers in elementary school about the initial symptoms of EDs, such as preoccupations indicative of body image dissatisfaction, can help as part of expanding public policy related to these aspects of adolescent health.
